# Does the Addition of a Lateral Extra-articular Procedure to a Primary Anterior Cruciate Ligament Reconstruction Result in Superior Functional and Clinical Outcomes? A Systematic Review and Meta-analysis of Randomized Controlled Trials

**DOI:** 10.1177/03635465241304781

**Published:** 2025-01-27

**Authors:** Adrian Kan, Tayla English, Allanah Penny, Jordan Franc-Smith, Francois Tudor, Larissa Sattler

**Affiliations:** † Bond Institute of Health and Sport, Robina, Australia; ‡ Orthopaedic Clinics Gold Coast, Gold Coast, Australia; Investigation performed at the Bond Institute of Health and Sport, Bond University, Robina, Australia

**Keywords:** anterior cruciate ligament reconstruction, lateral extra-articular procedure, rotatory instability, graft rupture

## Abstract

**Background:**

Current research focused on clinical outcomes suggests that lateral extra-articular procedures (LEAPs) can reduce rotational instability and graft failure rates in primary anterior cruciate ligament reconstructions (ACLRs). Limited studies have investigated the functional outcomes after LEAPs, including patient-reported outcome measures, sports participation, and physical performance.

**Purpose:**

To conduct a systematic literature review and meta-analysis to determine whether the addition of a LEAP to an ACLR results in superior functional and clinical outcomes as compared with an isolated ACLR.

**Study Design:**

Systematic review and meta-analysis; Level of evidence, 1.

**Methods:**

Five databases were searched to identify randomized controlled trials comparing clinical and functional outcomes after the addition of LEAPs to an isolated primary ACLR. Study selection was performed in accordance with the PRISMA guidelines (Preferred Reporting Items for Systematic Reviews and Meta-analyses). Assessment of methodological quality for included studies was undertaken using the Cochrane Risk of Bias 2 tool for randomized controlled trials. Studies were eligible for meta-analysis if an outcome measure utilizing similar time points was present across ≥2 studies and reported in mean difference or standard deviation.

**Results:**

Meta-analysis of 10 studies showed that the addition of LEAPs to an ACLR can reduce rates of rotatory instability (risk ratio, 1.45 [95% CI, 1.17-1.79]; *P* = .0006; *I*^2^ = 0%) and graft rupture (risk ratio, 0.21 [95% CI, 0.08-0.55]; *P* < .001; *I*^2^ = 0%). As supported by studies eligible for meta-analysis, this review showed that the addition of LEAPs to an ACLR can reduce rotatory instability. Short-term morbidity, including increased pain, joint stiffness, and muscle weakness, as compared with isolated ACLRs was resolved by 12 months after surgery.

**Conclusion:**

ACLR in combination with a LEAP results in superior clinical outcomes when compared with an isolated ACLR. Despite early postoperative outcomes concerning pain and function favoring isolated ACLRs, any negative effects were not still observed 6 months after surgery. A conclusion around the correlation between LEAPs and accelerated knee osteoarthritis could not be drawn, owing to the lack of long-term prospective studies available.

In line with the rising number of injuries to the anterior cruciate ligament (ACL) of the knee, rates of ACL reconstruction (ACLR) are increasing, with 400,000 ACLR procedures performed in the United States annually.^22,27,38^ Despite the relative success of ACLR in improving functional outcomes, up to 25% of patients with ACLR experience ongoing anterolateral instability during clinical examination after surgery,^28,29,58^ and a further 6% sustain a secondary ipsilateral rupture within 2 years owing to graft failure.^47^ Several strategies to mitigate these complications have been explored, such as optimizing tunnel positioning, varying graft and fixation options, and, increasingly, the addition of a lateral extra-articular procedure (LEAP) to primary ACLR in higher-risk populations.

Benefits of the addition of LEAPs, such as a lateral extra-articular tenodesis (LET) or an anterolateral ligament reconstruction, are greater rotational control and reduced secondary graft failure rates.^4,13,25^ These improved outcomes may be attributed to the anterolateral complex of the knee, including peripheral structures such as the iliotibial band and the anterolateral ligament, which act as a secondary restraint to anterior displacement and internal rotation of the tibia.^1,30,39^ The clinical advantages observed through the addition of a LEAP to ACLR, when compared with primary ACLR in isolation, are supported by improved self-reported function and higher return-to-sport rates, particularly in younger populations.^5,17,44^ However, despite the benefits of LEAPs, concerns remain regarding increased short-term postoperative pain and the risk of overconstraining the lateral tibiofemoral compartment, thus leading to degenerative changes, specifically with respect to the nonanatomic LET procedure.^7,9,40^

To date, research investigating the efficacy of adding a LEAP to primary ACLR appears to focus predominantly on clinical outcomes, including pivot-shift grade and secondary graft rupture.^12,40,44,46^ Yet, some uncertainty remains, particularly in terms of patient-reported functional outcomes and pain intensity when comparing the benefits of adding a LEAP.^12,40,44,46^ Therefore, the primary aim of this study was to conduct a systematic literature review and meta-analysis of randomized controlled trials to determine whether the addition of a LEAP to an ACLR results in superior functional and clinical outcomes, without increasing complications, as compared with a stand-alone ACLR.

## Methods

### Study Design

A systematic review was performed according to the PRISMA guidelines (Preferred Reporting Items for Systematic Reviews and Meta-analyses),^45^ and the study protocol was prospectively registered with Open Science Framework (doi.org/10.17605/OSF.IO/PHFVC).

### Data Sources and Search Strategy

A systematic search was conducted from inception through September 7, 2023, using the following databases: PubMed, Embase, CINHAL, Cochrane, and SPORTDiscus. Key terms included “anterior cruciate ligament” OR “ACL” AND “anterolateral ligament” OR “extra-articular” AND “tenodesis” OR “reconstruction” OR “augmentation.” Full search strategies for each database are presented in Appendix 1. All records were imported into EndNote software for screening.^11^

### Eligibility Criteria

Studies meeting the PICOS criteria were included in this review:

Population: individuals who underwent primary ACLR surgery
Intervention: primary ACLR surgery with the addition of a LEAP
Comparison: isolated primary ACLR surgery
Outcomes of interest: instability, stiffness, rerupture rates, pain, satisfaction, and physical function
Study design: randomized controlled trial

Exclusion criteria were as follows: revision ACLR surgery, use of nonhuman subjects, a study design other than a randomized controlled trial, and unavailable full text.

### Study Selection

After the removal of duplicates, 3 authors (T.E., J.F.-S., A.P.) independently performed a title and abstract screen, followed by a full-text screen in accordance with the eligibility criteria. Studies that did not meet the eligibility criteria were removed, with the reason provided. Discrepancies in the screening process were resolved by a fourth reviewer (L.S.), who facilitated group consensus agreement.

### Methodological Quality of Studies

Assessment of methodological quality was carried out independently by 2 authors (J.F.-S., A.P.) using the Cochrane Risk of Bias 2 tool for randomized controlled trials.^57^ Structured into 5 domains of bias, the Risk of Bias 2 tool provides a framework for assessing the risk of bias in a single result from individual studies to provide a judgment (low, some concerns, or high). A third author (T.E.) was consulted to achieve consensus in the instance of disagreement. After the methodological quality appraisal of each study, the kappa coefficient of interrater reliability was calculated, with values ranging from near perfect (0.81-1.00) to substantial (0.61-0.80), moderate (0.41-0.60), fair (0.21-0.40), and slight (0.0-0.2).

### Data Extraction

Study characteristics, participant description, surgical techniques, outcome measures, and results of studies were extracted independently by 3 authors (J.F.-S., T.E., A.P.). These results were recorded in a standardized data extraction table adapted from the Cochrane Collaboration data collection form.^23^

### Synthesis and Statistical Analysis

Meta-analysis was carried out for eligible studies using Review Manager software.^48^ Studies were eligible if an outcome measure utilizing similar time points was present across ≥2 studies and reported in mean differences or standard deviation. Confidence intervals from the outcome results were entered in the RevMan calculator to calculate the standard deviation if it was not provided. The heterogeneity of study outcome was determined via the *I*^2^ index, with values from 75% to 100% indicating considerable heterogeneity.^23^ Values are considered statistically significant when *P* < .05. Data not meeting the requirements of meta-analysis were synthesized and are discussed in narrative form.

## Results

### Selection of Sources of Evidence

**Figure 1.**
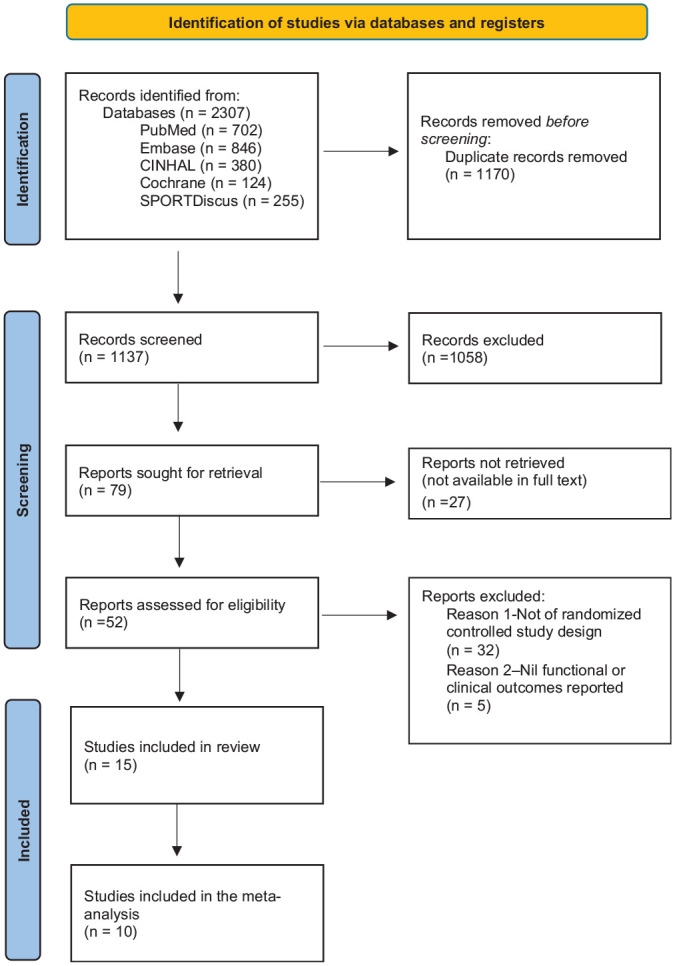
Flow diagram based on PRISMA (Preferred Reporting Items for Systematic Reviews and Meta-analyses).

Of the 2307 records identified in the initial search, 1137 remained after duplicate removal. After the screening process, 52 records were included for full-text review, with 15 randomized controlled trial reports remaining eligible for inclusion in this review. Per the *Cochrane Handbook for Systematic Reviews of Interventions* and the PRISMA guidelines, 10 of our studies were eligible for meta-analysis. The results of the literature search and screening process are summarized in Figure 1.

### Methodology Assessment

**Figure 2.**
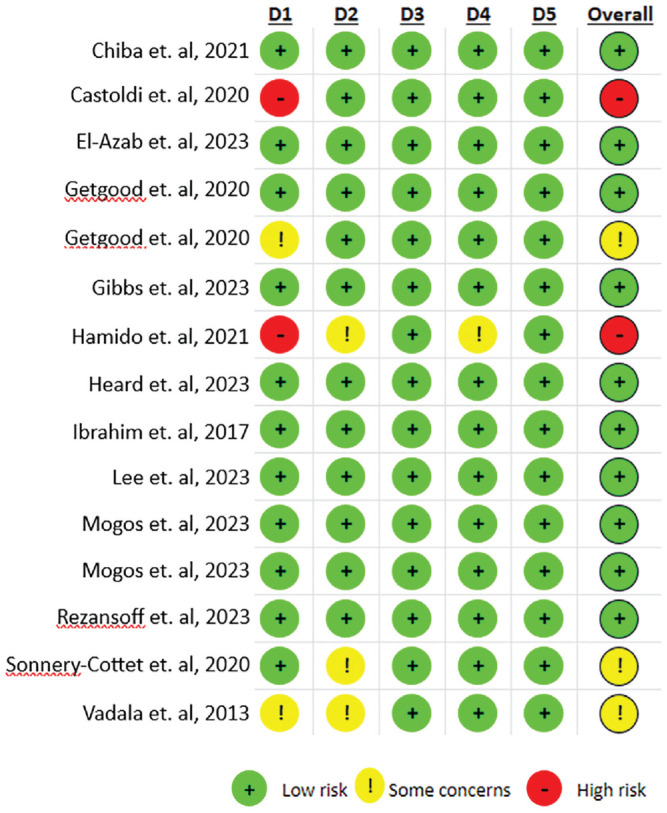
Risk of Bias 2 assessment. D1, randomization process; D2, deviations from the intended interventions; D3, missing outcome data; D4, measurement of the outcome; D5, selection of the reported result.

The Cochrane risk-of-bias assessment for all 15 studies is shown in Figure 2. Upon assessment, 10 studies were classified as low risk of bias,^§^ 3 studies presented some concern,^15,56,59^ and 2 studies were deemed to be of high risk of bias.^7,18^ This was predominately due to high risk within the randomization process and some concerns surrounding deviations from intended interventions. Four articles with some concern or high risk of bias^15,18,56,59^ found a significant difference in outcome measures of interest; however, 8 articles^10,14,16,21,24,36,37,49^ with low risk of bias also reported on these outcomes, therefore improving the quality of evidence. There was good interrater reliability between researchers, with the kappa coefficient equaling 0.80, which is regarded as substantial agreement.^33^

### Descriptive Participant Data

The 15 studies evaluated 1549 individuals with ACL tears. Overall, 513 males (66%) were included in the ACLR and ACLR + LEAP groups. Females were represented in 10 of the 15 studies, with 262 (34%) and 261 (34%) included in the ACLR and ACLR + LEAP groups, respectively. The mean age of patients was 26.3 years (range, 17-34) for ACLR and 25.5 years (range, 19-30) for ACLR + LEAP. Further details on descriptive characteristics for each study can be found in Table 1. All studies had a minimum postoperative follow-up period of 12 months. The inclusion criteria for all studies were an ACL-injured/deficient knee and high-grade pivot shift or rotational instability. Eight studies also had an inclusion criterion of participation in high-risk pivoting sports.^14,15,18,21,36,37,49^ Exclusion criteria varied among studies; however, the presence of multiligament injury, previous ACLR, or symptomatic cartilage defect requiring treatment other than debridement were commonly seen. The inclusion and exclusion criteria for all studies are presented in Appendix 2.

**Table 1:** Participant Description^*a*^

		Control			Experimental			
First Author (Year)	Country	No. of Participants	Sex, M:F	Mean Age, y	No. of Participants	Sex, M:F	Mean Age, y	Follow-up, mo (Range)
Castoldi (2020)^7^	France	61	43:18	25.9	60	47:13	26.2	228
Chiba (2021)^8^	USA	9	6:3	18.9	9	5:4	22	12
El-Azab (2023)^10^	Egypt and Austria	48	41:7	27	47	39:8	28	24
Getgood (2020)^14^	Canada	172	79:93	18.7	169	75:94	19	6, 12, 24
Getgood (2020)^15^	Canada	312	151:161	18.8	306	151:155	19.1	3, 6, 12, 24
Gibbs (2023)^16^	USA, Munich, Japan	7	4:3	17.9	7	4:3	23	6, 12
Hamido (2021)^18^	Kuwait	54	54:0	26	53	53:0	24	60 (55-65)
Heard (2023)^21^	Canada and Europe	312	151:161	18.8	306	151:155	19.1	3, 6, 12, 24
Ibrahim (2017)^24^	Kuwait	52	52:0	26	50	50:0	24	60 (55-65)
Lee (2023)^34^	Korea	36	31:5	33.4	38	33:5	30.9	27 (24-40)
Mogos (2023)^36^	Italy and Romania	26	17:9	34	32	26:6	28.8	1.3, 2.8, 6, 12, 24
Mogos (2023)^37^	Italy and Romania	25	17:8	33.8	32	27:5	28.8	1.3, 2.8, 6, 12, 24
Rezansoff (2024)^49^	Canada and Europe	285	139:146	18.8	268	128:140	18.8	6, 12, 24
Sonnery-Cottet (2020)^56^	France	112	96:16	25.9	112	78:34	24.7	12, 24
Vadalà (2013)^59^	Italy	32	0:32	27	28	0:28	27	44.6 (36-50)

a F, female; M, male.

### Surgical Characteristics

Of the studies, LET was performed in 9 stud-ies,^7,8,10,14-16,21,49,59^ while an anterolateral ligament reconstruction procedure was performed in the remaining 6 studies.^18,24,34,36,37,56^ Bone–patellar tendon–bone and quadriceps tendon grafts were utilized exclusively in 2 studies^7,56^ and 1 study,^18^ respectively, while 1 study used a mixture of bone–patellar tendon–bone and quadriceps tendon grafts.^16^ Semitendinous grafts were used in the remaining 11 studies.^∥^ For the LET procedures, 8 of the 9 studies used the modified Lemaire technique,^7,8,10,14-16,21,49^ with 1 study using the modified Cocker-Arnold technique.^59^ LET graft selection varied among iliotibial band,^8,14-16,21,49,59^ gracilis,^7^ and a combined semitendinous-gracilis,^10^ and the mean LEAP graft size was 10 mm in diameter × 80 to 100 mm long. All 6 studies that utilized anterolateral ligament reconstruction passed the gracilis graft deep to the iliotibial band; however, studies reported different attachment points, such as the femoral epicondyle, femoral condyle, Gerdy tubercle, and fibula head.^18,24,34,36,37,56^ Further details regarding surgical characteristics can be found in Table 2.

**Table 2:** Surgical Characteristics^*a*^

		LEAP	
LEAP Type: Author (Year)	ACL Reconstruction Graft	Graft	Graft Size, mm: Length × Width/Diameter
**LET**			
Castoldi (2020)^7^	BTB	Gracilis	150 × NR
Chiba (2021)^8^	Quadriceps	ITB	80-100 × 10
El-Azab (2023)^10^	Semitendinosus-gracilis	Semitendinosus-gracilis	NR
Getgood (2020)^14^	Semitendinosus-gracilis, Semitendinosus	ITB	80 × 10
Getgood (2020)^15^	Semitendinosus-gracilis, Semitendinosus	ITB	80 × 10
Gibbs (2023)^16^	BTB or quadriceps autograft	ITB	80 × 10
Heard (2023)^21^	Semitendinosus-gracilis, Semitendinosus	ITB	80 × 10
Rezansoff (2024)^49^	Semitendinosus and gracilis	ITB	80 × 10
Vadalà (2013)^59^	Semitendinosus and gracilis	ITB	80 × 10
**ALL**			
Hamido (2021)^18^	Semitendinosus	Gracilis	100 × 4.4-5.5
Ibrahim (2017)^24^	Semitendinosus	Gracilis	30 × 4-5
Lee (2023)^34^	Semitendinosus and gracilis^*b*^ and semitendinosus^*b*^	Gracilis	NR
Mogos (2023)^36^	Semitendinosus and gracilis	Gracilis	NR
Mogos (2023)^37^	Semitendinosus and gracilis	Gracilis	NR
Sonnery-Cottet (2020)^56^	BTB	Semitendinosus and gracilis	NR

a ACL, anterior cruciate ligament; ALL, anterolateral ligament; BTB, bone–patellar tendon–bone; ITB, iliotibial band; LEAP, lateral extra-articular procedure; LET, lateral extra-articular tenodesis; NR, not reported.

b Tibialis graft used for ACL reconstruction if patients preferred allograft.

### Outcome Measures

Patient-reported outcome measures were used to assess pain intensity, knee symptoms, quality of life, and sport-recreation participation, while physical performance and clinical outcomes were determined via rotatory instability and adverse events such as graft rupture. Summarized results of individual studies are presented in Table 3 with the full results presented in Appendix 3.

**Table 3:** Outcome Measures^*a*^

LEAP Type: Author (Year)	Self-reported Symptoms and Function	Pain	QoL and Sport Participation	Clinical Outcomes	Physical Performance	Graft Rupture
**LET**						
Castoldi (2020)^7^	=			×		
Chiba (2021)^8^	=			✓		
El-Azab (2023)^10^				✓		✓
Getgood (2020)^14^	=	=			=	
Getgood (2020)^15^	=	=	=	✓		✓
Gibbs (2023)^16^				✓		
Heard (2023)^21^		=			=	✓
Rezansoff (2024)^49^	=	=	=	=	×	✓
Vadalà (2013)^59^	=	=		✓		
**ALL**						
Hamido (2021)^18^	✓			✓		
Ibrahim (2017)^24^	=			✓		
Lee (2023)^34^	=	=	=	=		=
Mogos (2023)^36^	=			✓	=	✓
Mogos (2023)^37^	✓			✓		=
Sonnery-Cottet (2020)^56^	✓	=	✓	=	=	=

a ✓, favors ACLR + LET; ×, favors ACLR in isolation; =, no difference. ACL, anterior cruciate ligament reconstruction; ALL, anterolateral ligament; LEAP, lateral extra-articular procedure; LET, lateral extra-articular tenodesis; QoL, quality of life.

### Self-reported Function

**Figure 3.**
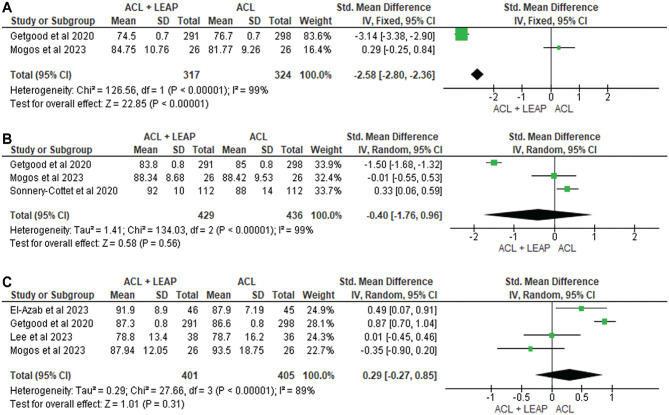
Meta-analysis comparing International Knee Documentation Committee of the ACL reconstruction + LEAP versus isolated ACL reconstruction at (A) 6 months, (B) 12 months, and (C) 24 months. ACL, anterior cruciate ligament; LEAP, lateral extra-articular procedure.

**Figure 4.**
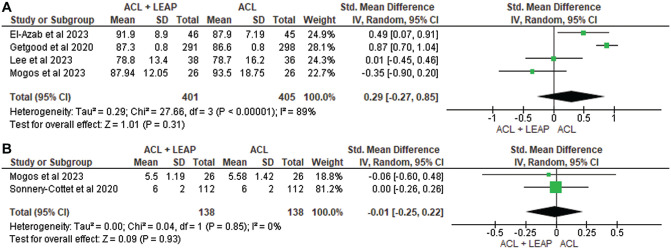
Meta-analysis comparing Tegner of the ACL reconstruction + LEAP versus isolated ACL reconstruction at (A) 12 months and (B) 24 months. ACL, anterior cruciate ligament; LEAP, lateral extra-articular procedure.

**Figure 5.**
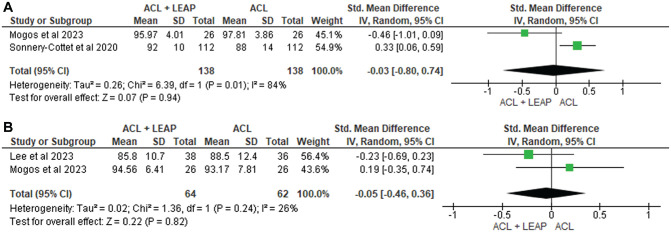
Meta-analysis comparing Lysholm of the ACL reconstruction + LEAP versus isolated ACL reconstruction at (A) 12 months and (B) 24 months. ACL, anterior cruciate ligament; LEAP, lateral extra-articular procedure.

Self-reported function was measured in 12 studies^¶^ through the International Knee Documentation Committee (IKDC) questionnaire, Lower Extremity Functional Scale, Knee injury and Osteoarthritis Outcome Score (KOOS), Lysholm Score, Forgotten Knee Score, Tegner Activity Scale, and Marx Activity Score. At 6 months, through pooled analysis (Figure 3), there was a significant difference in favor of the ACLR + LEAP group for the IKDC (standardized mean difference [SMD], −2.58 [95% CI, −2.80 to −2.36]; *P* < .001; *I*^2^ = 99%). No significant difference between groups was found at 12 months in the IKDC^14,36,56^ (SMD, −0.40; [95% CI, −1.76 to 0.96] *P* = .56; *I*^2^ = 99%) (Figure 3), Tegner^36,56^ (SMD, −0.01 [95% CI, −0.25 to 0.22] *P* = .93; *I*^2^ = 0%) (Figure 4), or Lysholm^36,56^ (SMD, −0.03 [95% CI, −0.80 to 0.74] *P* = .94; *I*^2^ = 84%) (Figure 5). At 24 months, no significant difference occurred in the IKDC^10,14,34,36^ (SMD, –0.29 [95% CI, −0.27 to 0.85]; *P* = .31; *I*^2^ = 89%), Tegner^36,56^ (SMD, −0.01 [95% CI, −0.25 to 0.22]; *P* = .93; *I*^2^ = 0%), or Lysholm^34,36^ (SMD, −0.05 [95% CI, −0.46, 0.36]; *P* = .82; *I*^2^ = 26%). One study^14^ noted worse Lower Extremity Functional Scale scores in the ACLR + LEAP group at 6 months, although this was resolved by 12 months. The remaining 8 studies^7,8,24,34,36,37,49,59^ revealed no difference in self-reported function between groups.

### Pain

**Figure 6.**
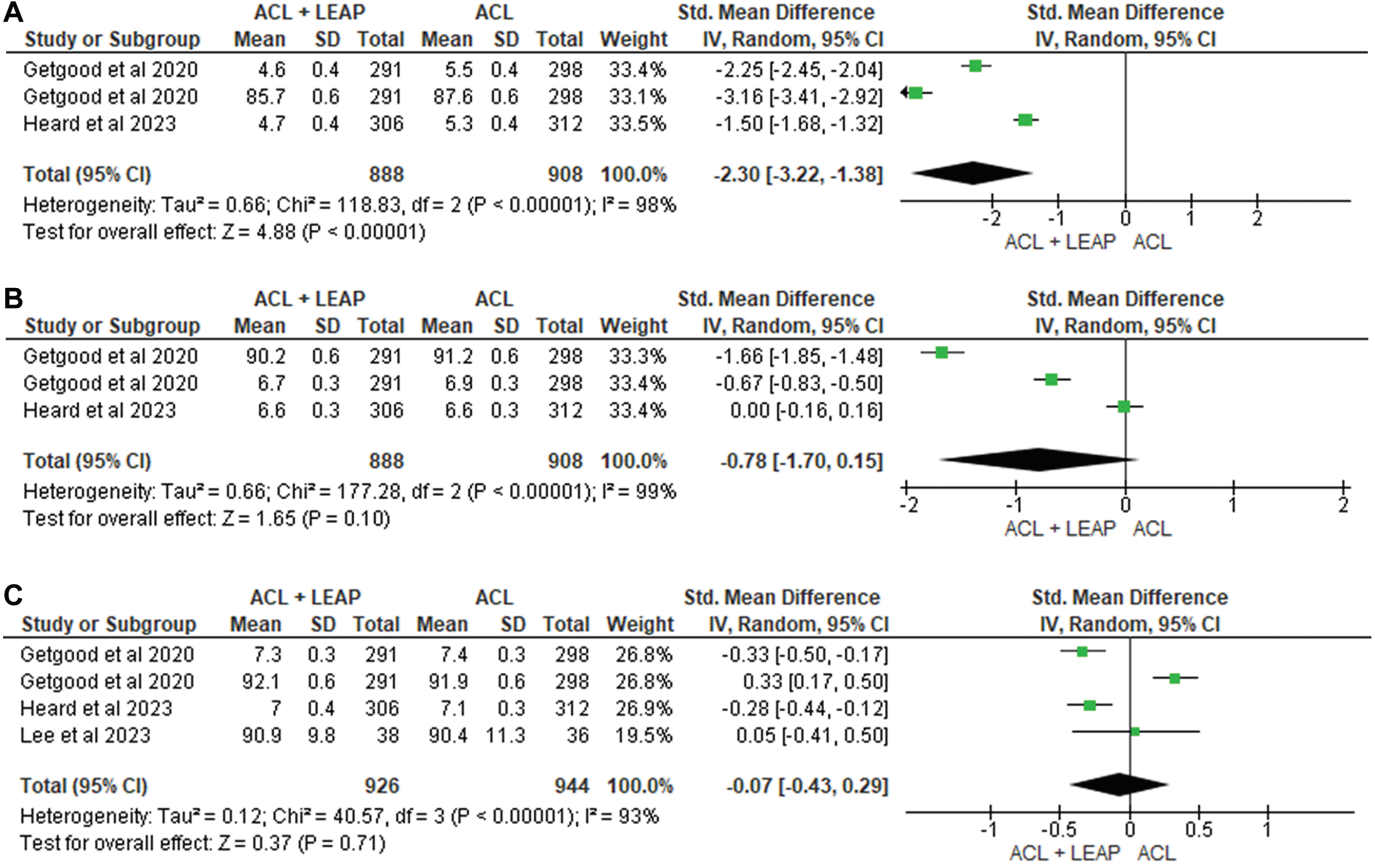
Meta-analysis comparing pain of the ACL reconstruction + LEAP versus isolated ACL reconstruction at (A) 6 months, (B) 12 months, and (C) 24 months. ACL, anterior cruciate ligament; LEAP, lateral extra-articular procedure.

Pain was assessed using the 4-item Pain Intensity Measure, visual analog scale, and KOOS. Of the 8 studies^8,14,15,21,34,49,56,59^ investigating pain, 3 reported significant increases in pain in the ACLR + LET group at 3 months^14,15,21^ and 1 at 6 months^15^ after surgery. Three eligible studies^14,15,21^ were pooled for analysis at 6 months after surgery, finding a significant increase in pain in those undergoing the ACLR + LET procedure (SMD, −2.30 [95% CI, −3.22, −1.38]; *P* < .0001; *I*^2^ = 98%) (Figure 6). However, at 12 months^14,15,21^ (SMD, −0.78 [95% CI, −1.70 to 0.15]; *P* = .10; *I*^2^ = 99%) and 24 months^14,15,21,34^ (SMD, −0.07 [95% CI, −0.43, 0.29]; *P* = .71; *I*^2^ = 93%), studies yielded no significant difference. The remaining 5 also revealed no difference between groups.^8,34,49,56,59^

### Quality of Life and Sport-Recreation Participation

Quality of life was measured through the KOOS and ACL Quality of Life questionnaire. While 1 study reported improved scores in the ACLR + LET group at 12 months after surgery,^56^ another noted poorer scores in the ACLR + LET group at 3 and 6 months.^15^ A study that specifically measured sports participation found no significant difference between groups at 24 months, with 76% of participants who previously played high-risk sports returning to them.^49^

### Clinical Outcomes

**Figure 7.**
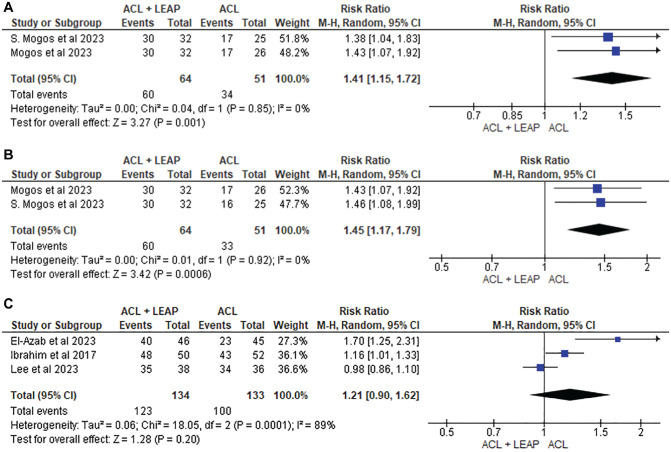
Meta-analysis comparing pivot shift grade 0 in ACL reconstruction + LEAP versus isolated ACL reconstruction at (A) 6 months, (B) 12 months, and (C) 24 months. ACL, anterior cruciate ligament; LEAP, lateral extra-articular procedure.

Clinical outcomes were included in 13 of 15 studies.^#^ Outcomes included osteoarthritis (OA) progression, Lachman test, pivot-shift test, recurrent instability, side-to-side difference of anterior tibial translation and tibial rotation, anterior drawer test, KT-1000/KT-2000 arthrometer, and Rolimeter differential anterior laxity. Castoldi et al^7^ noted an increase in the presence of OA in the lateral tibiofemoral compartment in the ACLR + LET group when compared with the ACLR-only group, although this study was of low quality. At 6 months, the side-to-side difference of anterior tibial translation of the ACLR + LET group was greater than that of the ACLR-only group, although this was undetected at 12-month follow-up.^8^ Of the 11 studies that measured pivot shift, 6 cited improved results in those who underwent an ACLR with LEAP^15,18,24,36,37,59^ when compared with isolated ACLR. This was supported by the 2 studies^36,37^ eligible for pooled analysis, which reported significant reductions in pivot shift at 6 months (risk ratio [RR], 1.41 [95% CI, 1.15-1.72]; *P* = .001; *I*^2^ = 0%) and 12 months (RR, 1.45 [95% CI, 1.17-1.79]; *P* = .0006; *I*^2^ = 0%) (Figure 7). No significant difference was found at 24 months (RR, 1.21 [95% CI, 0.90-1.62]; *P* = .20; *I*^2^ = 89%).^10,24,34^ There were no significant differences between groups for the Lachman, recurrent instability, anterior drawer, KT-1000/KT-2000 arthrometer, or Rolimeter differential anterior laxity.

### Physical Performance

Physical performance was measured using muscle strength, including quadriceps and hamstring peak torque and mean power, knee range of motion, limb symmetry index, and single-leg hop test. Five studies assessed physical performance,^14,21,36,49,56^ with two finding a decrease in quadriceps strength in the ACLR + LET group—one at 6 months and the other at the point of return to sport.^14,49^ One study revealed a decrease in passive extension and active assisted flexion in the ACLR + LET group at 3 months after surgery.

### Graft Rupture and Other Adverse Events

**Figure 8.**
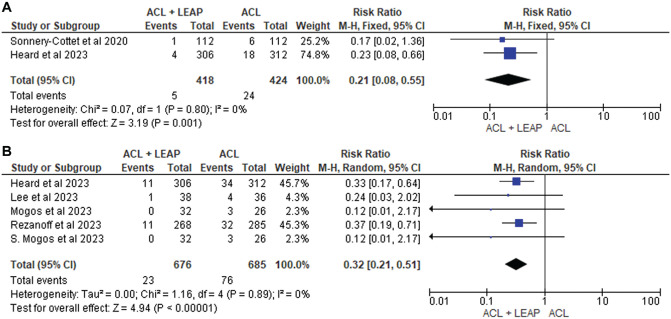
Meta-analysis comparing graft rupture of the ACL reconstruction + LEAP versus isolated ACL reconstruction at (A) 12 months and (B) 24 months. ACL, anterior cruciate ligament; LEAP, lateral extra-articular procedure.

Graft rupture after surgery was assessed by 8 articles,^10,15,21,34,36,37,49,56^ with 5 finding significantly lower graft rupture rates in the ACLR + LET group at 24 months.^10,15,21,36,49^ This was supported by 5 of the 8 studies^21,34,36,37,49^ eligible for pooled analysis (Figure 8), which reported a significantly lower graft rupture rate at 24 months after surgery (RR, 0.32 [95% CI, 0.21-0.51]; *P* < .001; *I*^2^ = 0%). At 12 months, 2 studies^21,56^ were pooled and similarly revealed a significantly reduced risk of graft rupture in the ACLR + LEAP group (RR, 0.21 [95% CI, 0.08-0.55]; *P* = .001; *I*^2^ = 0%).

## Discussion

The results of this review support the addition of a LEAP to a primary ACLR as compared with isolated ACLR. In line with past literature,^2,19,35,44,63^ our review reports reductions in joint instability and, thus, graft rupture rates with improved return to sports. Despite a short-term increase in pain, joint stiffness, and muscle weakness after LEAPs, this was no longer detected by 6 months after surgery.

Previous literature has demonstrated that LEAPs restore internal tibial rotation^56^ and can significantly reduce tibial acceleration during pivot-shift tests.^19^ This is consistent with current findings, where 6 of the 11 studies^10,15,18,36,37,59^ measuring changes to pivot-shift testing at 6 months reported significant improvements after the addition of LEAPs as compared with isolated ACLRs. This is supported by our meta-analysis, which revealed favorable outcomes at 6 and 12 months (Figure 7). Increased joint stability may be attributed to the function of the LEAP acting as a secondary stabilizer to the ACL by reducing anterior tibial translation and internal tibial rotation.^31^ This was evident in biomechanical studies utilizing cadaveric knees, which noted a decrease in internal rotation.^3^ As seen in this review, of which 5 studies were eligible for pooled analysis (Figure 8), the restoration in joint stability after LEAPs can minimize excessive strain on the ACLR graft, thus significantly reducing the risk of rupture up to 24 months after surgery.^21,34,36,37,49^

In the findings of this review, the lack of added benefit for rotatory stability after LEAPs to primary ACLR at 24 months after surgery may be explained by the accepted time frame for ACL graft incorporation, which is typically achieved by 24 months.^26,51^ Furthermore, strength gains through a structured rehabilitation program potentially provide stability similar to that of a graft reinforced by LEAPs. Although improvements in rotatory stability after LEAPs were seen only within the first 24 months after surgery, it could be argued that LEAPs are still beneficial given that the risk of sustaining an ACL graft rupture is the highest within 12 months after reconstruction.^52^ Conversely, changes to anterior laxity—as assessed via Lachman, anterior drawer, and arthrometer, regardless of time frame—were not seen in this review, with the exception of 1 study,^56^ which demonstrated that ACL with anterolateral ligament reconstruction led to a decrease in anterior laxity by 7.3 mm.

Increased pain after LEAPs as compared with isolated ACLRs remains a concern.^35,44,53^ As seen in 3 of the studies,^14,15,21^ which were eligible for pooled analysis (Figure 6), a significant increase in pain after the addition of LEAPs was noted within the first 6 months. Subsequent to additional surgical incisions and hardware irritation,^21,35,44^ the increase in postoperative pain may lead to reduced clinical outcomes, patient satisfaction, and physical function.^14,15,21^ However, it should be noted that short-term elevated pain levels after LEAPs were not detected at 6 months onward. This is consistent with the Marshall et al^35^ study, which reported that pain specific to LEAPs progressively resolves for 98.3% of patients within 12 months after surgery. Nonetheless, with a suggested 21.5% of patients requiring implant removal because of persistent pain after LEAPs,^35^ this may seem to suggest that patients with high levels of preoperative pain and increased pain sensitivity may not be appropriate candidates for LEAPs.^55^

Although a greater decrease in quadriceps strength and knee range of motion after LEAPs was reported within the first 6 months after surgery, this may be explained by increased short-term muscle weakness secondary to pain inhibition and muscle wastage^6,20,50^ caused by additional incisions. However, no between-group differences were seen at 12 months, with the exception of 1 study,^49^ which noted muscle strength to be significantly lower after LEAPs at the time of return to sport. Conversely, the increased joint stiffness may be due to the combined factor of greater swelling, inflammation, and potential for arthrofibrosis.^35^ On the contrary, the greater increase in self-reported function in favor of LEAPs as compared with isolated ACLRs at 6 months in our meta-analysis (Figure 3) may be a result of biomechanical changes that translate into self-reported function, such as knee stability.^32^

Given that the addition of LEAPs can reduce graft rupture by 2 to 3 times when compared with an isolated ACLR,^32,37,56^ it can be argued that the risk of short-term increases in pain and reduction in physical function is reasonably acceptable. However, concerns remain regarding the increased risk of degenerative changes and osteoarthritic progression in the long term.^7^ As a consequence of overconstraining the lateral compartment, the increased tibiofemoral contact pressure after LEAPs, especially during internal rotation, may overload the joint cartilage and thus cause osteoarthritic changes over time.^7,41,42,60^ While 1 of the studies^7^ reported increased tibiofemoral knee OA risk after LEAPs as compared with isolated ACLR, Sonnery-Cottet et al^56^ cited nil correlation between the factors. This is supported by past radiographic^9,62^ and kinematic studies.^43,61^ The inconsistency in findings and duration of study protocols indicates that a conclusion between increased knee OA risk and LEAPs cannot be drawn; therefore, future long-term prospective research in this area is needed.

### Strengths and Limitations

The strengths of this review include methodological rigor, adherence to PRISMA guidelines, and the inclusion criterion for a randomized controlled trial study design, resulting in a high level of evidence being reported.^54^ However, several limitations should be noted. Despite an increase in overall incidence of ACL ruptures seen among adolescents aged 5 to 14 years^63^ and the female population, 4 studies in this review excluded individuals aged <18 years,^10,16,34,56^ with male sex being dominant in the population. Therefore, a gap in the literature remains surrounding the benefits of LEAPs as compared with isolated ACLRs within higher-risk populations. Furthermore, clinical heterogeneity within studies—attributed to differences in follow-up duration, LEAP procedure type, fixation methods, graft type, and postoperative rehabilitation protocols—means that conclusions on the addition of LEAPs to isolated ACLRs in the longer term were difficult to draw.

## Conclusion

This review provides high-level evidence to support superior outcomes for a primary ACLR in combination with a LEAP when compared with an isolated ACLR for some clinical measures only. Despite early postoperative outcomes around pain and function favoring isolated ACLRs, these negative effects were no longer reported at 6 months after surgery. Conclusions around the correlation between LEAPs and accelerated knee OA could not be drawn at this time. Therefore, in line with past studies, this review supports the addition of LEAPs to primary ACLR, particularly for those at higher risk of graft failure. Further long-term prospective research investigating LEAPs with various graft types is needed.

## Supplemental Material

sj-pdf-1-ajs-10.1177_03635465241304781 – Supplemental material for Does the Addition of a Lateral Extra-articular Procedure to a Primary Anterior Cruciate Ligament Reconstruction Result in Superior Functional and Clinical Outcomes? A Systematic Review and Meta-analysis of Randomized Controlled Trials Supplemental material, sj-pdf-1-ajs-10.1177_03635465241304781 for Does the Addition of a Lateral Extra-articular Procedure to a Primary Anterior Cruciate Ligament Reconstruction Result in Superior Functional and Clinical Outcomes? A Systematic Review and Meta-analysis of Randomized Controlled Trials by Adrian Kan, Tayla English, Allanah Penny, Jordan Franc-Smith, Francois Tudor and Larissa Sattler in The American Journal of Sports Medicine

sj-pdf-2-ajs-10.1177_03635465241304781 – Supplemental material for Does the Addition of a Lateral Extra-articular Procedure to a Primary Anterior Cruciate Ligament Reconstruction Result in Superior Functional and Clinical Outcomes? A Systematic Review and Meta-analysis of Randomized Controlled Trials Supplemental material, sj-pdf-2-ajs-10.1177_03635465241304781 for Does the Addition of a Lateral Extra-articular Procedure to a Primary Anterior Cruciate Ligament Reconstruction Result in Superior Functional and Clinical Outcomes? A Systematic Review and Meta-analysis of Randomized Controlled Trials by Adrian Kan, Tayla English, Allanah Penny, Jordan Franc-Smith, Francois Tudor and Larissa Sattler in The American Journal of Sports Medicine

sj-pdf-3-ajs-10.1177_03635465241304781 – Supplemental material for Does the Addition of a Lateral Extra-articular Procedure to a Primary Anterior Cruciate Ligament Reconstruction Result in Superior Functional and Clinical Outcomes? A Systematic Review and Meta-analysis of Randomized Controlled Trials Supplemental material, sj-pdf-3-ajs-10.1177_03635465241304781 for Does the Addition of a Lateral Extra-articular Procedure to a Primary Anterior Cruciate Ligament Reconstruction Result in Superior Functional and Clinical Outcomes? A Systematic Review and Meta-analysis of Randomized Controlled Trials by Adrian Kan, Tayla English, Allanah Penny, Jordan Franc-Smith, Francois Tudor and Larissa Sattler in The American Journal of Sports Medicine

## Footnotes

§ References 8, 10, 14, 16, 21, 24, 34, 36, 37, 49.

∥ References 10, 14, 15, 18, 21, 24, 34, 36, 37, 49, 59.

¶ References 7, 8, 14, 15, 18, 24, 34, 36, 37, 49, 56, 59.

# References 7, 8, 10, 15, 16, 18, 24, 34, 36, 37, 49, 56, 59.
