# Supplementary material for: Does the Addition of a Lateral Extra-articular Procedure to a Primary Anterior Cruciate Ligament Reconstruction Result in Superior Functional and Clinical Outcomes? A Systematic Review and Meta-analysis of Randomized Controlled Trials
Source: Am J Sports Med. 2025 Jan 27;53(11):2749–60. doi: 10.1177/03635465241304781 (PMC12381392; doi:10.1177/03635465241304781)
Supplement: sj-pdf-1-ajs-10.1177_03635465241304781 – Supplemental material for Does the Addition of a Lateral Extra-articular Procedure to a Primary Anterior Cruciate Ligament Reconstruction Result in Superior Functional and Clinical Outcomes? A Systematic Review and Meta-analysis of Randomized Controlled Trial [file sj-pdf-1-ajs-10.1177_03635465241304781.pdf]

Database	Search Strategy
PubMed	("Anterior Cruciate Ligament"[mesh]) OR (ACL[Title/Abstract])) AND ("anterolateral ligament"[Title/Abstract] OR extraarticular [Title/Abstract] OR extra-articular[Title/Abstract] OR "extra articular"[Title/Abstract])) AND (tenodesis[Title/Abstract] OR reconstruction[Title/Abstract] OR augmentation[Title/Abstract])
EmBase	('Anterior Cruciate Ligament'/exp OR ACL: ti,ab) AND ('anterolateral ligament':ti,ab OR extraarticular:ti,ab OR extra-articular:ti,ab OR 'extra articular':ti,ab) AND (tenodesis:ti,ab OR reconstruction:ti,ab OR augmentation:ti,ab)
CINHAL	((((MH "Anterior Cruciate Ligament+")) OR ((TI ACL OR AB ACL))) AND ((TI "anterolateral ligament" OR AB "anterolateral ligament") OR (TI extraarticular OR AB extraarticular) OR (TI extra-articular OR AB extra-articular) OR (TI "extra articular" OR AB "extra articular"))) AND ((TI tenodesis OR AB tenodesis) OR (TI reconstruction OR AB reconstruction) OR (TI augmentation OR AB augmentation))
Cochrane	([mh "Anterior Cruciate Ligament"]) OR (ACL:ti,ab)AND ("anterolateral ligament":ti,ab OR extraarticular:ti,ab OR extra-articular:ti,ab OR "extra articular":ti,ab) AND (tenodesis:ti,ab OR reconstruction:ti,ab OR augmentation:ti,ab)
SportsDiscus	(((DE "Anterior Cruciate Ligament") OR ((TI "ACL" OR AB "ACL"))) AND ((TI "anterolateral ligament" OR AB "anterolateral ligament") OR (TI "extraarticular" OR AB "extraarticular") OR (TI "extra-articular" OR AB "extra-articular") OR (TI "extra articular" OR AB "extra articular"))) AND ((TI "tenodesis" OR AB "tenodesis") OR (TI "reconstruction" OR AB "reconstruction") OR (TI "augmentation" OR AB "augmentation"))
