# Supplementary material for: Does the Addition of a Lateral Extra-articular Procedure to a Primary Anterior Cruciate Ligament Reconstruction Result in Superior Functional and Clinical Outcomes? A Systematic Review and Meta-analysis of Randomized Controlled Trials
Source: Am J Sports Med. 2025 Jan 27;53(11):2749–60. doi: 10.1177/03635465241304781 (PMC12381392; doi:10.1177/03635465241304781)
Supplement: sj-pdf-2-ajs-10.1177_03635465241304781 – Supplemental material for Does the Addition of a Lateral Extra-articular Procedure to a Primary Anterior Cruciate Ligament Reconstruction Result in Superior Functional and Clinical Outcomes? A Systematic Review and Meta-analysis of Randomized Controlled Trial [file sj-pdf-2-ajs-10.1177_03635465241304781.pdf]

Author, Year & Country	Title	Inclusion	Exclusion
M. Castoldi et al. 2020 France	A Randomized Controlled Trial of Bone-Patellar Tendon-Bone Anterior Cruciate Ligament Reconstruction With and Without Lateral Extra-articular Tenodesis: 19-Year Clinical and Radiological Follow-up	• Complete isolated primary ACL rupture with a plan for arthroscopic ACLR.	• History of ACL repair or reconstruction<br>• Associated tears of the posterior cruciate ligament<br>• Injuries of the collateral ligaments requiring surgical treatment.
D. Chiba et al. 2021 USA	Lateral Extra-articular Tenodesis Contributes Little to Change In Vivo Kinematics After Anterior Cruciate Ligament Reconstruction: A Randomized Controlled Trial	• Rotatory knee instability (>3mm lateral translation or side-side difference of >50%) evaluated by PIVOT technology and inertial sensor with the patient under general anesthesia<br>• Contralateral healthy knee	Not specified

		without any history of surgery, within 12 months from the ACL injury<br>• No greater than grade 2 injury to MCL or LCL, absence of PCL injury	
H. El-Azab et al. 2023 Egypt and Austria	A comparison of the outcomes of anterior cruciate ligament reconstruction with large-size graft versus reconstruction with average-size graft combined with extraarticular tenodesis	• ACL deficient knee was clinically manifested by physical examination and confirmed by MRI.<br>• Age 18-45 years, skeletally mature patient.<br>• Positive pivot shift of at least grade 2 required	• Multiple ligament injuries or a polytraumatized patient<br>• Revisions or an ACL tear in the opposite knee.<br>• Generalized laxity.<br>• Symptomatic articular cartilage defect requiring treatment; Outerbridge > grade II

			• >3 degrees of varus or valgus malalignment
A. Getgood et al.<br>2020<br>Canada	No Difference in Functional Outcomes When Lateral Extra-Articular Tenodesis Is Added to Anterior Cruciate Ligament Reconstruction in Young Active Patients: The Stability Study	• ACL deficient knee requiring surgical construction<br>• Skeletally mature<br>• No older than 25 years at the time of surgery<br>• Had 2 or more of the following:<br>- Participated in a competitive pivoting sport<br>- Had a positive pivot shift grade of 2 or higher	• Undergone previous ACL reconstruction on either knee<br>• Required bilateral ACL reconstruction<br>• Required surgical repair or reconstruction of PCL, LCL, MCL or posterolateral corner<br>• Symptomatic articular cartilage defect requiring treatment other than debridement

		- Had generalized ligament laxity or genu recurvatum greater than 10 degrees	• Greater than 3 degrees of asymmetric varus alignment
A. M. J. Getgood et al.<br>2020<br>Canada	Lateral Extra-articular Tenodesis Reduces Failure of Hamstring Tendon Autograft Anterior Cruciate Ligament Reconstruction: 2-Year Outcomes From the STABILITY Study Randomized Clinical Trial	• Between 14 - 25 years old<br>• ACL deficient knee<br>• Higher risk of reinjury based on the presence of 2 or more:<br>- participation in competitive pivoting sports<br>- grade 2 pivot shift or greater<br>- generalized ligament laxity or genu	• Previous ACLR on either knee<br>• Multiligament injury<br>• Symptomatic articular cartilage defect requiring treatment other than debridement<br>• Greater than 3 degrees of asymptomatic varus<br>• Unable or unwilling to be

		recurvatum greater than 10 degrees	followed for 2 years postoperatively<br>• Skeletally immature
C. M. Gibbs et al. 2023 USA, Munich, Japan	Preoperative quantitative pivot shift does not correlate with in vivo kinematics following ACL reconstruction with or without lateral extraarticular tenodesis	• Sustained ACL injury within 12 months without contralateral knee injury<br>• Between 18-40yrs<br>• High Grade rotational instability (pivot = or >3mm	• >/= grade 2 collateral ligament injury<br>• Cartilage injury<br>• Those not remaining local for 1year post-op<br>• Pregnant
F. Hamido et al. 2021 Kuwait	Anterolateral ligament reconstruction improves the clinical and functional outcomes of anterior cruciate ligament reconstruction in athletes	• High grade pivot shift (3)<br>• Segond fracture<br>• High level of sports activity<br>• Frequent pivoting sports	• History of knee surgery<br>• Knee dislocation<br>• Pre-op OA

			• Revision ACLR<br>• Multiligament knee injury
M. Heard et al. 2023<br>Canada & Europe	No increase in adverse events with lateral extra-articular tenodesis augmentation of anterior cruciate ligament reconstruction - Results from the stability randomized trial	• Within age range<br>• ACL deficient knee<br>• Higher risk of re-injury<br>• Two or more of the following:<br>- Participating in competitive pivoting sport<br>- Presence of grade 2 pivot shift or greater<br>- generalized ligament laxity<br>- genu recurvatum greater than 10deg	• Any of the following present:<br>- Previous ACLR on either knee<br>- Multiligament injury<br>- Symptomatic articular cartilage defect requiring more than debridement<br>- Greater than 3 deg asymmetric varus<br>- unable or unwilling to be followed-up for 2 years post

S. A. Ibrahim et al. 2017<br>Kuwait	Anatomic Reconstruction of the Anterior Cruciate Ligament of the Knee With or Without Reconstruction of the Anterolateral Ligament: A Randomized Clinical Trial	• Unilateral ACL tear<br>• Indications used for combined method:<br>-Grade 2 pivot<br>-High level sporting<br>-Pivoting sports<br>-Chronic ACL injury<br>-Segond fracture	• Revision ACL<br>• multi-ligament injury
S. S. Lee et al. 2023<br>Seoul	Single Bundle Anterior Cruciate Ligament With Anterolateral Ligament Reconstruction Yields Similar Clinical and Radiographic Results at Minimum Two-Year Follow-Up Versus Double Bundle Anterior Cruciate Ligament Reconstruction: A Prospective Randomized Controlled Trial	• ACL tear<br>• Underwent DB ACL or combined SB+ ALLR<br>• Aged between 18 -60yrs<br>• No history of contralateral ACL reconstruction<br>• Follow up of more than 2 years	• Revision ACLR<br>• Concomitant ligament surgeries

Ș. Mogos et al. 2023<br>Italy & Romania	Superior rotational stability and lower re-ruptures rate after combined anterolateral and anterior cruciate ligament reconstruction compared to isolated anterior cruciate ligament reconstruction: a 2-year prospective randomized clinical trial	• Presence of a unilateral primary ACL tear in skeletally mature patients<br>• Preoperative pivot shift test grade 2 or 3<br>• Participation in high grade pivoting sports<br>• Chronic ACL lesion	• Recurrent ACL tears<br>• Previous surgery of affected knee<br>• Presence of multiligament injuries of knee<br>• Necessity to associate an extra-articular procedure (other than ALLR) or posterior cruciate ligament reconstruction<br>• Rheumatoid arthritis

Ş. Mogoş et al. 2023<br>Italy and Romania	Combined Anterior Cruciate Ligament and Anterolateral Ligament Reconstruction Results in Superior Rotational Stability Compared with Isolated Anterior Cruciate Ligament Reconstruction in High Grade Pivoting Sport Patients: A Prospective Randomized Clinical Trial	• Unilateral primary ACL tear<br>• Participation in high-grade pivoting sport<br>• Preoperative pivot shift test grade II or III	• Recurrent ACL tears<br>• Previous surgery of affected knee<br>• Presence of multiligament injuries of knee<br>• Necessity to associate an extra-articular procedure (other than ALLR) or posterior cruciate ligament reconstruction<br>• Rheumatoid arthritis<br>• Skeletal maturity not reached

A. Rezansoff et al.<br>2023<br>Canada & Europe	Anterior Cruciate Ligament Reconstruction Plus Lateral Extra-articular Tenodesis Has a Similar Return to Sports Rate as ACLR Alone, But Lower Failure Rate	• Aged between 15-25 years<br>• ACL deficient knee<br>• High-risk of re-injury (defined as presence of 2 or more of following:<br>1) participant in competitive pivoting sport<br>2) presence of grade 2 pivot shift or greater<br>3) generalized ligament laxity (Beighton score of 4 or greater) or genu recurvatum >10deg	• Previous ACLR on either knee<br>• Multiligament injury<br>• Symptomatic articular cartilage defect requiring treatment other than debridement<br>• Greater than three degrees of asymmetric varus<br>• Unable or willing to be followed for up to 2 years postop<br>• Patients who preoperatively participated in low-risk sports or no sports

B. Sonnery-Cottet et al. 2020<br>France	Combined ACL and Anterolateral Reconstruction Is Not Associated With a Higher Risk of Adverse Outcomes: Preliminary Results From the SANTI Randomized Controlled Trial	• ACL deficiency requiring ACLR<br>• Patient aged 18-35 years old	• Multiligament deficiency<br>• Previous ligament surgery on affected or contralateral knees<br>• Body mass index <18.5 or >30kg/square m<br>• Professional athletes<br>• Connective tissue disorder or congenital disease<br>• Refusal to take part in the study<br>• Chondral lesions requiring treatment
A. P. Vadalà et al. 2013<br>Italy	An extra-articular procedure improves the clinical outcome in anterior cruciate ligament reconstruction with hamstrings in female athletes	• Presence of a moderate to severe rotational instability as revealed by a pivot-shift test graded as 2+ or 3+<br>• Minimum interval of 2 months	• Previous surgical procedures on same or contralateral knee<br>• Concomitant injury of internal or external collateral ligament<br>• Concomitant systemic diseases

	between trauma and surgery<br>• Age<40 years old	• pre-operative signs of knee arthritis<br>• Imaging evidence of grades III or IV chondral damage on both patella surface or medial and lateral femoral condyles
