# Supplementary material for: Does the Addition of a Lateral Extra-articular Procedure to a Primary Anterior Cruciate Ligament Reconstruction Result in Superior Functional and Clinical Outcomes? A Systematic Review and Meta-analysis of Randomized Controlled Trials
Source: Am J Sports Med. 2025 Jan 27;53(11):2749–60. doi: 10.1177/03635465241304781 (PMC12381392; doi:10.1177/03635465241304781)
Supplement: sj-pdf-3-ajs-10.1177_03635465241304781 – Supplemental material for Does the Addition of a Lateral Extra-articular Procedure to a Primary Anterior Cruciate Ligament Reconstruction Result in Superior Functional and Clinical Outcomes? A Systematic Review and Meta-analysis of Randomized Controlled Trial [file sj-pdf-3-ajs-10.1177_03635465241304781.pdf]

Author, Year	Outcome Measure (s)	Measurement Tool	Timepoint of Assessment	P value (<0.05)
Castoldi et. al, 2020	1. Clinical outcomes<br>2. Patient reported outcome measures.	1a. Medial tibiofemoral OA<br>1b. Lateral tibiofemoral OA<br>1c. Patellofemoral OA<br>1d. Lachman's Test<br>1e. Pivot Shift Test<br>2a. IKDC<br>2b. Lysholm Score<br>2c. Forgotten Knee Score<br>2d. Tegner Activity Score	19 years	1a. NS<br>1b. 0.0001^<br>1c. NS<br>1d. NS<br>1e. NS<br>2a. NS<br>2b. NS<br>2c. NS<br>2d. NS
Chiba et. al, 2021	1. Clinical outcomes<br>2. Patient reported outcome measures.	1a. SSD of ATT<br>1b. SSD of TR<br>2a. Marx Activity Score<br>2b. KOOS	6 months<br>12 months	6 months<br>1a. 0.03*<br>1b. NS<br>2a. NS<br>2b. NS<br><br>12 months<br>1a. NS<br>1b. NS

				2a. NS<br>2b. NS
El-Azab et. al, 2023	1. Clinical outcomes<br>2. Patient reported outcome measures	1a. Pivot shift test<br>2a. IKDC	2 years	1a. Reported as significant with no p value.<br>2a. NS
Getgood et. al, 2020	1. Patient reported outcome measures.<br>2. Physical performance	1a. Four-item pain intensity measure<br>1b. Lower Extremity Functional scale<br>2a. Limb Symmetry Index<br>2b. Quadricep index peak torque<br>2c. Quadriceps average power<br>2d. HTI peak torque<br>2e. HTI average power<br>2f. HTI/QI ratio	3 months<br>6 months<br>12 months<br>24 months	3 months<br>1a. <0.05^<br>1b. 0.01^<br>2a. NM<br>2b. NM<br>2c. NM<br>2d. NM<br>2e. NM<br>2f. NM<br><br>6 months<br>1a. NS<br>1b. 0.02^<br>2a. NS

2b. 0.03^

2c. 0.01^

2d. NS

2e. 0.05^

2f. NS

12 months

1a. NS

1b. NS

2a. NS

2b. NS

2c. NS

2d. NS

2e. NS

2f. NS

24 months

1a. NS

1b. NM

2a. NS

				2b. NS<br>2c. NS<br>2d. NS<br>2e. NS<br>2f. NS
Getgood et.al, 2020	1. Clinical outcomes<br>2. Patient reported outcome measures.<br>3. Adverse events	1a. Pivot shift<br>2a. Four-item pain intensity scale<br>2b. Marx activity rating scale<br>2c. ACL quality of life questionnaire<br>2d. IKDC<br>2e. KOOS Pain<br>2f. KOOS Symptoms<br>2g. KOOS ADLs<br>2h. KOOS Recreation<br>2i. KOOS QOL<br>3a. Graft rupture shown by MRI	3 months<br>6 months<br>12 months<br>24 months	3 months<br>1a. NM<br>2a. 0.003^<br>2b. NM<br>2c. NM<br>2d. 0.01^<br>2e. 0.007^<br>2f. 0.005^<br>2g. 0.006^<br>2h. 0.01^<br>2i. 0.03^<br>3a. NM<br><br>6 months

1a. NM<br>2a. NS<br>2b. NM<br>2c. NM<br>2d. 0.03^<br>2e. 0.02^<br>2f. 0.002^<br>2g. 0.004^<br>2h. 0.02^<br>2i. 0.02^<br>3a. NS
12 months<br>1a. NM<br>1b. NM<br>2a. NS<br>2b. NM<br>2c. 0.01^<br>2d. NS

				2e. NS<br>2f. NS<br>2h. NS<br>2i. 0.02^<br>3a. NS<br><br>24 months<br>1a. 0.0001*<br>2a. NS<br>2b. NS<br>2c. NS<br>2d. NS<br>2e. NS<br>2f. NS<br>2g. NS<br>2h. NS<br>2i. NS<br>3a. 0.001*
Gibbs et. al, 2023	1. Clinical outcomes	1a. Quantitative pivot shift<br>1b. AP tibial translation	Pre-operative<br>6 months	6 months<br>1a. NS

		1c. TR	12 months	1b. NS<br>1c. NS<br><br>12 months<br>1a. NS<br>1b. <0.05*<br>1c. NS
Hamido et. al, 2021	1. Clinical outcomes<br>2. Patient reported outcome measures	1a. Pivot Shift<br>1b. Anterior drawer test<br>1c. Lachman's test<br>1d. KT-1000<br>2a. Lysholm Score<br>2b. IKDC<br>2c. Tegner Activity Score	Pre-operative<br>60 months	Pre-operative<br>1a. NS<br>1b. NM<br>1c. NM<br>1d. NS<br>2a. NS<br>2b. NS<br>2c. NS<br><br>60 months<br>1a. 0.001*<br>1b. NS<br>1c. NS

				1d. 0.001*<br>2a. NS<br>2b. 0.001*<br>2c. NS
Heard et. al 2023	1. Patient reported outcome measures.<br>2. Physical performance<br>3. Adverse events	1a. Four-item pain intensity scale<br>2a. ROM knee extension<br>2b. ROM knee flexion<br>3a. Minor medical adverse events<br>3b. Minor surgical events<br>3c. Contralateral ACL rupture<br>3d. Graft rupture	3 months<br>6 months<br>12 months<br>24 months	3 months<br>1a. 0.004^<br>2a. 0.03<br>2b. <0.01<br>3a. NM<br>3b. NM<br>3c. NM<br>3d. NM<br><br>6 months<br>1a. NS<br>2a. NS<br>2b. NS<br>3a. NM<br>3b. NM

3c. NM

3d. NM

12 months

1a. NS

2a. NS

2b. NS

3a. NM

3b. NM

3c. NM

3d. NM

24 months

1a. NS

2a. NS

2b. NS

3a. NS

3b. NS

3c. NS

3d. <0.01*

Ibrahim et. al, 2017	1. Clinical outcomes<br>2. Patient reported outcome measures	1a. Anterior Drawer Test<br>1b. Lachman's Test<br>1c. Pivot Shift Test<br>1d. KT-1000 arthrometer<br>2a. Lysholm Score<br>2b. Tegner activity score<br>2c. IKDC	Pre-operative<br>27 months	Pre-operative<br>1a. NM<br>1b. NM<br>1c. NS<br>1d. NS<br>2a. NS<br>2b. NS<br>2c. NS<br><br>27 months<br>1a. NS<br>1b. NS<br>1c. NS<br>1d. <0.001*<br>2a. NS<br>2b. NS<br>2c. NS
Lee et. al, 2023	1. Clinical outcomes	1a. Lachman's Test<br>1b. Pivot shift Test	Pre-operative<br>6-9 months	Pre-operative<br>1a. NS

2. Patient reported outcome measures<br>3. Adverse Events	1c. Anterior translation on radiographs<br>1d. KT-2000 arthrometer<br>1e. ROM<br>2a. Lysholm Score<br>2b. IKDC<br>2c. Tegner Activity Score<br>2d. KOOS Pain<br>2e. KOOS Symptoms<br>2f. KOOS ADLs<br>2g. KOOS Recreation<br>2h. KOOS QOL<br>3a. Graft tear on MRI	1b. NS<br>1c. NS<br>1d. NS<br>1e. NS<br>2a. NS<br>2b. NS<br>2c. NS<br>2d. NS<br>2e. NS<br>2f. NS<br>2g. NS<br>2h. NS<br>3a. NM<br><br>6-9 months<br>1a. NS<br>1b. NS<br>1c. NS<br>1d. NS<br>1e. NS

				2a. NS<br>2b. NS<br>2c. NS<br>2d. NS<br>2e. NS<br>2f. NS<br>2g. NS<br>2h. NS<br>3a. NS
Mogos et. al,<br>2023	1. Clinical Outcomes<br>2. Patient reported outcome measures<br>3. Physical Performance<br>4. Adverse Events	1a. Lachman's Test<br>1b. Pivot Shift Test<br>1c. Rollimeter differential anterior laxity<br>2a. IKDC<br>2b. Lysholm Score<br>2c. Tegner Activity Score<br>3a. ROM<br>4a. Graft rupture	Pre-operative (T0)<br>6 weeks (T1)<br>12 weeks (T2)<br>6 months (T3)<br>12 months (T4)<br>24 months (T5)	Pre-operative<br>1a. NS<br>1b. NS<br>1c. NS<br>2a. NS<br>2b. NS<br>2c. NS<br>3a. NS<br>4a. NM<br><br>6 weeks

1a. NM<br>1b. 0.02*<br>1c. NS<br>2a. NS<br>2b. NS<br>2c. NS<br>3a. NS<br>4a. NM<br><br>12 weeks<br>1a. NM<br>1b. 0.03*<br>1c. NS<br>2a. NS<br>2b. NS<br>2c. NS<br>3a. NS<br>4a. NM<br><br>6 months

1a. NM<br><br>1b. 0.016*<br><br>1c. NS<br><br>2a. NS<br><br>2b. NS<br><br>2c. NS<br><br>3a. NS<br><br>4a. NM<br><br><br>12 months<br><br>1a. NM<br><br>1b. 0.016*<br><br>1c. NS<br><br>2a. NS<br><br>2b. NS<br><br>2c. NS<br><br>3a. NS<br><br>4a. NM<br><br><br>24 months

				1a. NM<br>1b. NM<br>1c. NM<br>2a. NS<br>2b. NS<br>2c. NS<br>3a. NS<br>4a. 0.04*
Mogos et. al,<br>2023	1. Clinical Outcomes<br>2. Patient reported outcome measures<br>3. Adverse events	1a. Pivot Shift Test<br>1b. Rollimeter differential laxity<br>2a. Subjective IKDC<br>2b. Objective IKDC<br>3a. Graft rupture	6 weeks<br>12 weeks<br>6 months<br>12 months	6 weeks<br>1a. 0.03*<br>1b. NS<br>2a. NS<br>2b. NS<br>3a. NS<br><br>12 weeks<br>1a. 0.007*<br>1b. NS<br>2a. NS<br>2b. NS

				3a. NS<br><br>6 months<br>1a. 0.007*<br>1b. NS<br>2a. NS<br>2b. 0.03*<br>3a. NS<br><br>12 months<br>1a. 0.007*<br>1b. NS<br>2a. 0.04*<br>2b. 0.015*<br>3a. NS
Rezansoff et. al<br>2023	1. Clinical outcomes<br>2. Patient reported outcome measures<br>3. Physical performance<br>4. Adverse events	1a. Pivot Shift Test<br>2a. Lower Extremity Functional Scale<br>2b. Four-item pain intensity measure<br>3a. Quadriceps peak torque<br>3b. Hamstrings peak torque	24 months	24 months<br>1a. NS<br>2a. NS<br>2b. NS<br>3a. 0.005^

	5. Sport Participation	3c. Single leg hop test		3b. NS
		4a. Graft rupture		3c. NS
		5a. Return to high-risk sport		4a. 0.004*
		5b. Return to low-risk sport		5a. NS
		5c. No participation		5b. NS
				5c. NS
		1a. Rollimeter anteroposterior laxity		1a. NS
		2a. Tegner Activity Score		2a. NS
		2b. KOOS symptoms		2b. NS
		2c. KOOS pain		2c. NS
	1. Clinical outcomes	2d. KOOS ADLs		2d. 0.02*
	2. Patient reported outcome	2e. KOOS Recreation		2e. NS
Sonnery-Cottet	measures	2f. KOOS QOL	Pre-operative	2f. 0.008*
et. al, 2020	3. Physical Performance	2g. Subjective IKDC	12 months	2g. NS
	4. Adverse events	2h. Lysholm Score		2h. 0.013*
		3a. ROM knee flexion		3a. NS
		3b. ROM knee extension		3b. NS
		4a. Graft rupture		4a. NS
		4b. Contralateral ACL rupture		4b. NS
		4c. Cyclops syndrome		4c. 0.012*

		4d. Secondary meniscal procedure		4d. NS
Vadala et. al, 2013	1. Clinical outcomes<br>2. Patient reported outcome measures	1a. Pivot shift Test<br>1b. Lachman's Test<br>1c. KT-1000<br>2a. Tegner Activity Score<br>2b. Lysholm Score<br>2c. Subjective IKDC<br>2d. Visual Analogue Scale	36 – 50 months	1a. 0.003*<br>1b. NS<br>1c. NS<br>2a. NS<br>2b. NS<br>2c. NS<br>2d. NS

** significant difference favors ACLR+LEAP, ^ favors ACLR, NS = not significant, NM = not measured*
